# An extension to the collisional model of the energetic cost of support qualitatively explains trotting and the trot–canter transition

**DOI:** 10.1002/jez.2268

**Published:** 2019-04-29

**Authors:** James R. Usherwood

**Affiliations:** ^1^ Structure and Motion Laboratory The Royal Veterinary College Hatfield UK

**Keywords:** canter, collision, gait, gallop, trot

## Abstract

The majority of terrestrial mammals adopt distinct, discrete gaits across their speed range. Though there is evidence that walk, trot and gallop may be selected at speeds consistent with minimizing metabolic cost (Hoyt and Taylor, 1981, *Nature*, *291*, 239–240), the mechanical causes underlying these costs and their changes with speed are not well understood. In particular, the paired, near‐simultaneous contacts of the trot is puzzling as it appears to demand a high mechanical work that could easily be avoided with distributed contacts, as with a “running walk” gait or “tolt.” Here, a simple condition is derived—a ratio including the pitch moment of inertia and back length—for which trotting is energetically advantageous because it avoids the energetic consequences of pitching. Pitching could also be avoided if the impulses from the legs were orientated through the center of mass. A range of idealized gaits is considered that achieve this zero‐pitch condition, and work minimization predicts a transition from trot to canter at intermediate speeds. This can be understood from the geometric principles of achieving a “pseudoelastic” collision with each impulse (Ruina et al., 2005, *J Theoretical Biol*, *14*, 170‐192). However, at high speeds, a transition back to trot is predicted that is not observed in nature.

## Research highlights

Trotting and cantering gaits can avoid pitching and associated mechanical work demands. A transition from trot to canter with increasing speed is correctly predicted; however, the account fails at higher speeds, predicting a return to trotting.

## Graphical abstract

Considering the mechanical consequences of pitching—or what it takes to avoid pitching—provides an energetic account for trotting, cantering, and the trot–canter transition

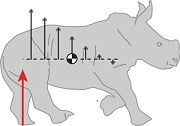

## 1 INTRODUCTION

Robert McNeill Alexander pioneered the application of simple conceptual and analytical models to questions of animal form and function especially relating to structure and gait. He advanced principles of work minimization while realizing simple physical and physiological constraints as a starting point for understanding many aspects of locomotor performance, from walking and running to leaping, quadrupedal gaits, and scaling of gaits. In this context, Alexander's approach may be considered highly reductionist. Not in terms of reducing to its simplest molecular or atomic scale, but in terms of conceptual reductionism. This paper continues to apply the reductionist approach in an attempt to understand the energetic implications of quadrupedal gaits.

Alexander's modeling based on work minimization of quadrupedal gaits encountered difficulty in accounting for both trotting and galloping, and a transition between the two with increasing speed (Alexander, 1988; Alexander, Jayes, & Ker, 1980; see also Xi, Yesilevskiy, & Remy, 2016). His account for galloping, therefore, focused on the potential role of elastic elements in the back to reduce the mechanical work associated with driving body motions with respect to the center of mass (see also Taylor, 1978; Yesilevskiy, Yang, & Remy, 2018). A more recent reductionist paradigm allowing consideration of work minimization in quadrupedal gaits has exploited the principles of collision mechanics (Ruina, Bertram, & Srinivasan, 2005). This has allowed focus on the energetic consequences of transitions between passive periods in the gait cycle—vaulting for walking or the ballistic aerial phase of running—providing (in retrospect) intuitive, work‐based accounts for such gait features as the shove‐crash strategy of stance transition in walking bipeds (Kuo, 2001; Ruina et al., 2005) and the merit of the distributed footfall of galloping (Ruina et al., 2005). Though the extreme form of this reduction, considering impulsive collisions imposed through massless legs acting on a point‐mass, has had some success and found some traction as an account for galloping gaits in the literature (e.g., Pfau, Witte, & Wilson, 2006), it has limitations. That is not to criticize it as a starting point; indeed, identifying the model failures allows focus on the next level of complexity required to account for features of gait.

One area where the most reduced impulsive approach fails is in accounting for trotting and quadrupedal gait transitions. The distributed footfall of galloping makes sense as a work‐minimizing strategy for a point‐mass body in a manner analogous to adding as many spokes as possible to a rolling rimless wheel. However, this approach fails to account for any gait that does *not* spread out the impulses into four contacts or “beats.” A “gathered” gallop, with impulses from hindlimbs followed quickly by impulses from fore before a longer ballistic phase with the legs “gathered” under the body, makes sense if excessive pitching is to be avoided by inclining the impulses somewhat toward the center of mass (CoM; Ruina et al., 2005). However, *why* do so many quadrupeds *ever* trot? Why use gaits with only two “beats” per stride cycle, with much higher CoM collisional losses (e.g., Lee et al., 2011)? The adoption of trotting at intermediate speeds might be considered the norm among terrestrial animals across a large size range, at least among terrestrial mammals. A few of the exceptions should be noted. Among larger terrestrial mammals, elephants (Hutchinson et al., 2006), giraffe (Dagg & Vos, 1968), wildebeest (Pennycuick, 1975), and adult brown bears (Shine, Penberthy, Robbins, Nelson, & McGowan, 2015) have been observed to not trot at intermediate speeds. Many smaller species (including rabbits and squirrels) also avoid trotting, but some do include a distinct trot in their repertoire (for instance the banded mongoose—personal observation).

Though near‐trotting footfall timing may have some benefit for highly sprawled animals in terms of allowing a geometric contribution from back flexion (Gray, 1968), there appears to be no current general and mechanistic account for the prevalence of trotting at intermediate speeds among upright mammals, nor for measured advantage (at suitable speeds) of trotting over walking or galloping in terms of metabolic energy consumption (Hoyt & Taylor, 1981).

## 2 APPROACHES AND OVERVIEW

The aim of this paper is to make simple, again reductionist, extensions to the collisional approach to provide an energetic account for trotting, and the transition from trotting to cantering with increasing speed, that might be sufficiently fundamental as to be viewed as general. The extensions proposed are: The energetic consequences of pitching due to hindlimbs and forelimbs connecting to a body behind and ahead of the center of mass; and the energetic consequences of avoiding this pitching.

This paper compares idealized gaits, with weight supported by momentary, impulsive forces provided by the limbs and acting at hip or shoulder girdle, with the center of mass located on a stiff back and positioned half way between hip and shoulder. It first compares idealized trotting—with simultaneous hind and fore impulses orientated vertically occurring twice per stride—with idealized tolting, with hind and fore vertical impulses spread evenly through time. This idealization of the tolt, a gait used by Icelandic horses, would also apply to the “rack” of other horses and “amble” of elephants and some primates. These gaits are perhaps best summarized as “running walks” as they have the same even phasing of limbs (hind–fore–hind–fore–hind…) as a normal horse walk; however, this term has unhelpful mechanical connotations. The term “tolt” is therefore adopted here, also because it appears to have greatest appropriate traction in the biomechanics literature. The idealized trot and tolt gaits allow the consequences of pitching with finite moments of inertia to be considered analytically with an analysis of energy fluctuations.

The paper continues by comparing five idealized gaits (tolt, trot, canter, gathered gallop, and even gallop) achievable with a negligible pitch moment of inertia such that the net orientation of every impulse or simultaneous impulses must pass through the center of mass. The energetic consequences of these impulses are calculated from collisional principles, largely following Ruina et al. (2005). This approach views the role of limb forces as a means of redirecting velocities, and reduces the forces to brief impulses or “collisions” providing a change in velocity with a brief (high) force acting in a single direction.

The effects of elasticity are not considered here. One particular line of thinking (e.g., O'Neill & Schmitt, 2012) should be addressed directly. It might be thought that the trot can be advantageous because it could allow relatively large elastic storage and return. But what is the energetic benefit of this loss‐recovery cycle, especially as it is not perfect? Would it not be better to select a gait, tolt perhaps, if it has a lower requirement for loss‐return cycling? Yes, less energy “saving” could be attributed to elastic return, but, given a matching hysteresis loss, the lost energy requiring “payment” through costly muscle work would be reduced. Elastic mechanisms may certainly play a role in reducing energetic demands of locomotion, but it is currently unclear whether, why or how hysteresis (due to proportional energy lost to heat) depends on gait. I, therefore, neglect elasticity here by assuming that its hysteresis is constant across all gaits, such that it is sufficient to only calculate the relative work demands of each gait; but I do acknowledge that this may be untrue (Alexander, 1988; Taylor, 1978).

## 3 WHY EVER TROT AND NOT TOLT?

The positive work associated with vertical impulses that are evenly spaced through time can be calculated for idealized tolting and trotting by calculating the fluctuations in kinetic energy due to vertical motions and rotational energies relating to pitching of the body. This comparison is valid for these two idealized gaits as both kinetic and rotational energies reach minima at the same moments, meaning that there is no opportunity for transfer between the two energy forms: The energy required to go from minimum vertical kinetic and rotational energy to maximum can be simply added.

The vertical velocity at the peak of each ballistic phase is zero. Ballistic equations of motion, therefore, give the vertical velocity due to gravity (of magnitude *g*) at the start of each ballistic phase *V_y_* (just after contact) as

(1) $$V_{y\text{,trot}}=\frac{{\mathrm{gT}}_{\text{stride}}}{4}$$

for two cycles per stride period *T*_stride_ in trotting, and

(2) $$V_{y\text{,tolt}}=\frac{gT_{\text{stride}}}{8}$$

for four cycles per stride in the tolt. The summed positive work required to provide kinetic energy to a body of mass *m* for vertical motions in a gravity of magnitude *g* is therefore

(3) $$\sum W_{y,\text{trot}}^{+}=2\left(\frac{1}{2}mV_{y,\text{trot}}^{2}\right)=2\left(\frac{1}{2}\frac{mg^{2}T_{\text{stride}}^{2}}{4^{2}}\right)=\frac{mg^{2}T_{\text{stride}}^{2}}{16}$$

for trot, and

(4) $$\sum W_{y,\text{tolt}}^{+}=\;4\left(\frac{1}{2}mV_{y,\text{tolt}}^{2}\right)=4\left(\frac{1}{2}\frac{mg^{2}T_{\text{stride}}^{2}}{8^{2}}\right)=\frac{mg^{2}T_{\text{stride}}^{2}}{32}$$

for tolt. This confirms the result (Ruina et al., 2005) that a gait with double the number of half the magnitude impulses can require, from a point center of mass perspective, half the work.

The tolt, however, alternates impulses from hind and front limbs. To oppose body weight over each stride, each of the four limbs imposes an impulse of magnitude $mgT_{\text{stride}}/4$ with a lever arm of approximately half the horizontal back (hip to shoulder) length. The change in angular velocity Δω of pitch of the body depends on the lever arm and body pitch moment of inertia (second moment of mass) *I*:

(5) $$\left|\Delta \omega \right|\;=\;\frac{mgT_{\text{stride}}}{4}\;\frac{L_{\text{back}}}{2}\;\frac{1}{I}.$$

With evenly timed impulses in tolt, the pitching reverses sense each stance, going past an instant of zero angular velocity at midstance, just as the vertical kinetic energy is also zero. The magnitude of angular velocity after the second half of stance is therefore $\left|\omega_{\text{max}}\right|=\frac{1}{2}\left|∆\omega \right|$. Given four impulses per stride cycle, the positive work required to provide rotational energy in pitch for tolt is

(6) $$\sum W_{\text{rot},\text{tolt}}^{+}\;=\;4\;\left(\frac{1}{2}I{\left|\omega_{\max}\right|}^{2}\right)\;=\;\frac{mg^{2}T_{\text{stride}}^{2}}{32}\frac{mL_{\text{back}}^{2}}{4I}.$$

Remembering the comparison being used of an idealized trot versus an idealized vertical impulse tolt, the three expressions for work can be combined to find the conditions under which trotting is energetically less costly than tolting.

(7) $$\sum W_{y,\text{trot}}^{+}\;<\;\sum W_{y,\text{tolt}}^{+}\;+\;\sum W_{\text{rot},\text{tolt}}^{+}$$

is true if

(8) $$\frac{4I}{mL_{\text{back}}^{2}}\;<1.$$

This derivation suggests a helpful normalization for the pitch moment of inertia,

(9) $$\hat{I}\;=\;\frac{4I}{mL_{\text{back}}^{2}},$$

with which mechanical work demands would be lower in the trot if back length and mass distribution resulted if $\hat{I}<1$.

## 4 CENTER OF PERCUSSION‐BASED DERIVATION

The energetic result above can be understood intuitively by relating it to the concept of the center of percussion. Does a vertical, upwards impulse *p* acting on the body at the hip accelerate the center of mass upwards (positive increase in $V_{y,\text{CoM}}$)? Yes, of course, as

(10) $$\Delta V_{y,\text{CoM}}=\frac{p}{m}.$$

**Figure 1:**
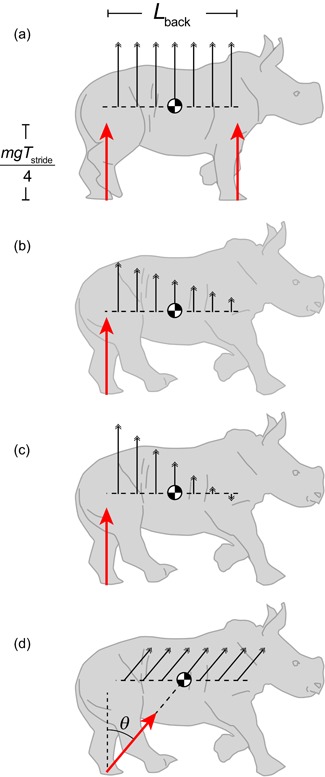
Potential acceleration consequences (black arrows) for a range of idealized cases, with impulses (red arrows) sufficient to support body weight over a stride. In the trotting idealization (a), impulses act vertically and simultaneously through hind and front legs, resulting in purely vertical acceleration at the center of mass and at all points along the back. In gaits (including tolt, canter and gallop) including unpaired stances, a vertical impulse results in pitching accelerations of the body about the center of mass (b,c). The unloaded girdle is accelerated upwards (the shoulder in [b]) if the pitch moment of inertia is above a critical value and tolting would be economical, or downward (c) if below this value, in which case trotting (avoiding the pitching) would be energetically superior. Unpaired stances do not result in pitching accelerations if their impulse vectors are directed through the center of mass (d) resulting in forward accelerations from unopposed hindlimbs and decelerations from unopposed forelimbs [Color figure can be viewed at wileyonlinelibrary.com]

But does it act to accelerate the shoulders up or down (Figure 1b,c)? This depends on the pitching acceleration. The pitching moment impulse, with the moment arm provided by half the back length, is −*pL*_back_/2 (i.e., nose‐down) and is resisted by the pitch moment of inertia *I*, resulting in a change in pitch angular velocity:

(11) $$\Delta \omega \;=\;-p\frac{L_{\text{back}}}{2}\;\frac{1}{I}.$$

The combined effect in terms of acceleration at the shoulder, located half a back length the other side of the CoM is then

(12) $$\Delta V_{y,\text{shoulder}}=\Delta V_{y,\text{CoM}}+\Delta \omega \frac{L_{\text{back}}}{2}=\frac{p}{m}-\frac{{pL}_{back}^{2}}{4I}.$$

Again, this can be arranged to show that the shoulder would be accelerated up if $\hat{I}>1$, down if $\hat{I}<1$, and not at all if $\hat{I}=1$, at which point the hips and shoulders are at their respective centers of percussion.

The implication of this in terms of stability of quadrupedal gaits based on spring‐like bouncing is clear (see Lee & Biewener, 2011 who acknowledge Murphy, 1984). If $\hat{I}\;>\;1$, a perturbation resulting in a slight increase in impulse at the hips *reduces* the impulse at the shoulders, thereby *increasing* the subsequent impulse at the hips… and so on; the system is unstable. For this reason, early quadrupedal robots and gait simulations seeking stability usually ensured $\hat{I}$<1 (e.g., Lee & Meek, 2005; Murphy, 1984).

However, the above analysis based on work demands shows a low $\hat{I}$, convenient for stability, comes at an energetic cost, even if trotting is adopted to remove any pitching.

As an aside, these analyses would also apply when comparing running with hopping, replacing back length with the width between hips, and pitch moment of inertia with roll moment of inertia: idealized hopping would be less costly in terms of mechanical work for a biped with broad hips and well separated foot placement and mass focused near to the center of mass. I do not explore this further here.

## 5 CHALLENGES TO MEASURING $\hat{I}$ IN AN ANIMAL

$\hat{I}$ is therefore an important metric when considering the energetic consequences of different quadrupedal gaits. However, it is difficult to either measure directly or calculate accurately: not only is it sensitive to small errors in length measures (as their effects are squared), it also assumes both moment of inertia and functional back length to be approximately constant throughout a stride and, for some comparisons, across gaits. These assumptions are clearly not valid for high‐speed locomotion in animals such as cheetah or greyhound. Despite preliminary efforts, I have not yet been able to determine convincingly whether $\hat{I}$ is indeed below one for animals that trot and above one for those mammals that never appear to.

## 6 ANALYSIS OF IDEALIZED, IMPULSIVE NONPITCHING GAITS

I proceed here by assuming that $\hat{I}$ is generally below one, such that trotting is economical in the case of purely vertical impulses. I consider the extreme case of $\hat{I}$ = 0, meaning that pitching moments cannot be resisted by inertia, and the combined effect of impulses acting at an instant must result in the net impulse vector passing through the center of mass. This can be achieved for a range of gaits if impulses from the hindlimbs are inclined sufficiently forward from vertical, by an angle *θ*, and accelerating aft‐fore impulses are opposed by forelimb impulses inclined similarly backwards. Idealized gaits (see Hildebrand, 1989 for basic kinematic gait descriptions) considered are:

*The trot*. Each stride has two ballistic phases and two pairs of synchronous hind–fore impulses. The net impulse vector originates between the hind and forefeet and is orientated vertically.

*The tolt*. Each stride has four ballistic phases evenly spaced through time, with alternating hind and fore impulses. Impulses therefore alternate between forward (from the hindlimbs) and backward (from the fore).

*The canter*. Each stride has a single ballistic phase finishing with a solo hind impulse (accelerating forwards) followed immediately by a paired hind–fore impulse acting vertically (as in the trot), followed immediately by a fore contact decelerating forward progression of the body. Two options are considered for the relative impulse magnitudes, the first used for the numerical model and the second to allow a simple analytical expression for good cantering conditions. The first option uses impulse magnitudes that result in a constant contribution to weight support from each limb (so the combined effect of the paired‐limb, middle vertical impulse is double that of the single limbs). The second assumes that there is an even magnitude of impulses between the three instants (hind, hind–fore pair, and fore). The conclusions are not sensitive to these assumptions.

*The gathered gallop*. Each stride has a single ballistic phase finishing with one forward‐inclined impulse from the first hind foot, immediately followed by a second identical impulse from the second hind foot, immediately followed by two backwards‐orientated impulses from the two forefeet.

*The even gallop*. Each stride has four evenly timed ballistic phases, similar to the tolt, but the two forward‐orientated (hindlimb) impulses follow each other, then come the two backward‐orientated impulses from the forelimbs. The analysis presented here is planar, and there is no consideration of whether the contacts are from left or right limbs. However, it is worth noting that the even gallop would be the closest model to a “rotary” gallop typical in high‐speed greyhound and cheetah, whereas the “gathered” gallop would be typical of a “transverse” gallop (see also Bertram & Gutmann, 2009; note that the rhino used in the figures happens to display a rotary footfall pattern; and that it repeatedly transitioned between the two patterns).

## 7 THE METHOD FOR CALCULATING THE WORK OF AN IMPULSE

**Figure 2:**
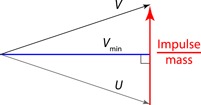
The geometry required in calculating the energetic consequences of a brief impulse resulting in a change of center of mass velocity from initial *U* to post‐impulse *V*. If the magnitude of the CoM velocity decreases, its kinetic energy falls and energy is dissipated. This loss continues until the minimum CoM velocity magnitude (at *V*_min_—which may also be *U* or *V*), after which, if the CoM velocity magnitude increases, kinetic energy is contributed. It is this positive energy that is considered the energetic work “cost” associated with the impulse. If the energy decreases and increases (*V*_min_ is less than *U* or *V*), the impulse may be viewed as resulting in a “pseudoelastic” collision—often a favorable condition, as this means forces are orientated close to perpendicular to velocities, thereby demanding low power and work for a given change in direction. CoM: center of mass [Color figure can be viewed at wileyonlinelibrary.com]

This is covered thoroughly elsewhere (e.g., Ruina et al., 2005) so is only summarized here. To calculate the work of each impulse, the center of mass velocity *U* immediately before the impulse of interest is accelerated by the impulse, resulting in a postimpulse velocity *V* (Figure 2). The changes in velocity vector through this, very brief, impulse are calculated numerically (100 divisions are used here; results are not sensitive to this; coded in LabVIEW, National Instruments, Austin, TX) to follow the progression of the center of mass velocity. When the magnitude of this velocity is decreasing, energy is being dissipated; when it increases, work is being applied to the CoM as kinetic energy. Only and all the positive work demand is calculated as costly. By presenting results as a proportion of the costs of trotting, adding a cost to negative work or reducing a cost due to elastic recoil (unless absolutely perfect) has no bearing on the results as long as these adjustments are not gait dependent.

Impulses are of sufficient magnitude to support body weight over a stride, and are orientated as described above. Incoming velocities are derived from ballistics and/or the condition after an immediately preceding impulse, and result in velocities providing initial conditions for a ballistic period or another impulse immediately following. Average horizontal velocity ${\bar{V}}_{x}$ is used to determine a dimensionless speed ${\hat{V}}_{x}$ following the principles set out by Alexander and Jayes (1983) concerning Froude number (but note the nonsquared form is adopted here), using leg length *L*_leg_ in the normalization:

(13) $${\hat{V}}_{x}=\frac{{\bar{V}}_{x}}{\sqrt{gL_{\text{leg}}}}.$$

Stride periods are normalized using ballistic (or pendular) principles (again see Alexander & Jayes, 1983) to give a dimensionless stride period:

(14) $$\hat{T_{\text{stride}}}=\frac{T_{\text{stride}}}{\sqrt{L_{\text{leg}}/g}}.$$

**Figure 3:**
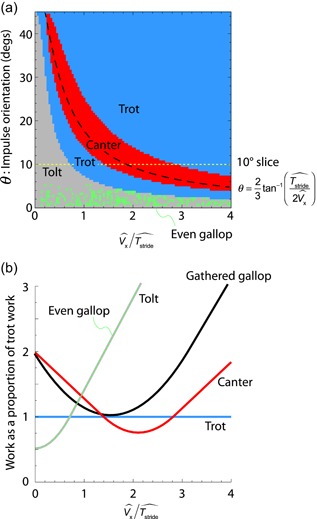
Results from the mechanical work calculations for a range of idealized impulsive gaits. The gaits minimizing work (a) vary according to a ratio of dimensionless speed and dimensionless stride period, and the orientation of single‐stance impulses where appropriate. At relatively low speeds, long stride periods, or upright impulses, tolting is energetically superior; with progressively higher speeds, briefer stride periods, or more inclined impulses, optimal gaits are predicted to transition from tolt to trot to canter… and back to trot. At low impulse inclinations, tolt and even gallop results converge so closely that computational limits are approached (hence the speckle). An analytical approximation for ideal conditions for canter is indicated (black dashed line). The yellow dotted line denotes the *θ* = 10° transect shown in (b) [Color figure can be viewed at wileyonlinelibrary.com]

It is helpful to note that the direction of the center of mass velocity at the instant of impulse depends on how many ballistic periods a stride cycle is divided into (determined by the gait) and the ratio of the dimensionless speed and stride period (used in Figure 3). Higher horizontal velocities, lower stride periods and more ballistic phases result in shallower (nearer horizontal) velocities at the end of each ballistic phase.

The idealized gaits considered here result in constant heights at the instant of each impulse.

## 8 RESULTS

The gait that minimizes mechanical work, of the idealized cases surveyed, is shown in Figure 3a. At low horizontal speeds (or large stride periods), tolting is superior at all impulse inclinations <45°. At intermediate speeds, stride periods, and impulse orientations, trotting is work‐minimizing. At some higher speed, lower stride period and/or higher impulse orientation, cantering is found to be work‐minimizing. This model of work minimization also predicts a further transition not towards galloping but instead back to trotting. This does not match observation in nature.

A slice of the cost surface taken at a 10° impulse orientation (Figure 3b) shows how the work cost of each gait compares with the trot as a function of speed (stride period). Tolt and even gallop values are nearly indistinguishable. Indeed, at higher speeds (lower stride periods) they get sufficiently close that selecting which is lower is subject to computational noise (hence the gray/green speckle, Figure 3a). This can be reduced with greater computational investment, but the phenomenon is useful in demonstrating that the work demands for tolt and even gallop may be functionally indistinguishable at any level relevant to physiological cost or selection pressure. The gathered gallop work approaches but never dips below that of the trot.

The predicted trot–canter transition does not, and was not expected to, provide a quantitative match with observation. Empirical impulse angles for canter are low (only around 6°; Merkens, Schamhart, van Osch, & Hartman, 1993), and predict a trot–canter transition speed about double that observed. However, the mechanical and energetic implications of the extremely idealized, reductionist gaits are revealing.

## 9 ACCOUNTING FOR CALCULATED ADVANTAGE OF TOLT AT VERY LOW SPEEDS

At sufficiently low speeds and small impulse inclinations, the energetic cost of horizontal fluctuations in velocity are small compared with the benefits of spreading the impulses across four instants per stride rather than two. The fact that the tolt‐trot transition is not commonly seen with increasing speed may be attributable to the omission of an idealized walk. In some respects, walk and tolt may be considered as similar gaits; however, the impulsive reduction as used here appears especially questionable for walk, for which finite stance durations and fore‐aft forces throughout the stride may be particularly relevant (e.g., Usherwood & Self Davies, 2017).

## 10 UNDERSTANDING THE PRINCIPLES UNDERLYING TROT–CANTER (–TROT) TRANSITION

**Figure 4:**
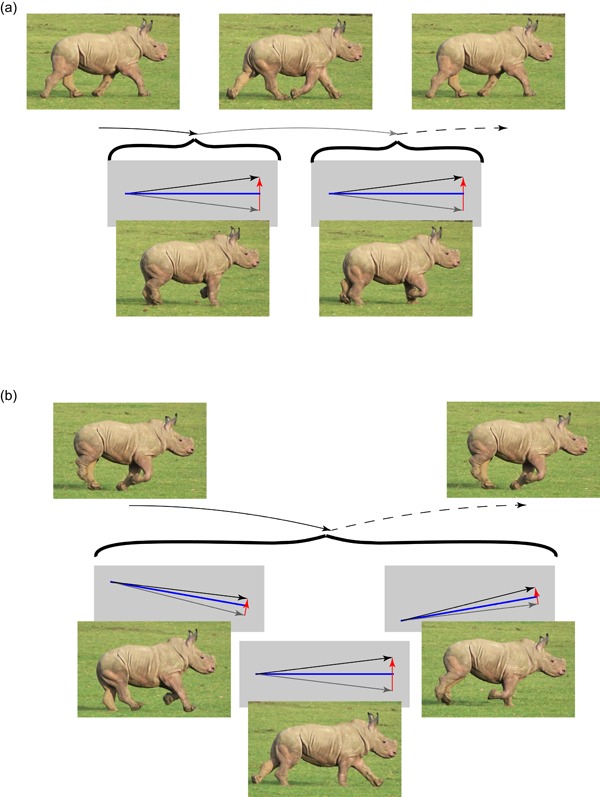
The geometry for trot (a) and canter (b) at $\hat{V_{\text{x}}}/\hat{T_{\text{stride}}}=2$, *θ* = 10° demonstrates why cantering is energetically superior under these conditions. Each momentary impulse (red arrow) changes the initial center of mass velocity (gray arrow) to a new vector (black arrow) leading into a ballistic period (trotting or the final impulse of cantering) or directly into the next impulse (first two impulses of cantering). Under these velocity, stride period and impulse inclination conditions, cantering enables redirection of the center of mass to be split among three pseudoelastic impulses rather than the two of trotting each stride; and each impulse is approximately perpendicular to *V*_min_ (blue lines), providing a smooth redirection of the CoM trajectory and resulting in both positive and negative work over each impulse. CoM: center of mass [Color figure can be viewed at wileyonlinelibrary.com]

**Figure 5:**
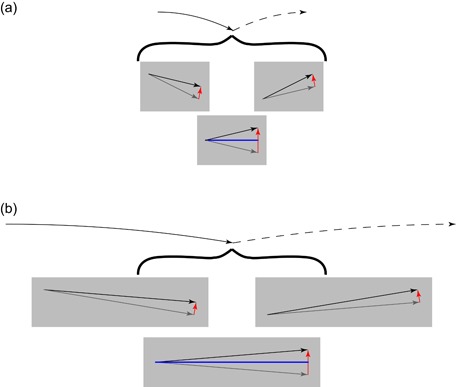
The geometries for canter at *θ* = 10° at (a) low ($\hat{V_{\text{x}}}/\hat{T_{\text{stride}}}=1$) or (b) high ($\hat{V_{\text{x}}}/\hat{T_{\text{stride}}}=3$) speeds. The impulses (red arrows) no longer act perpendicular to *V*_min_ during the first and third contacts. During the first impulse in the slow case, energy is only dissipated, demanding a high positive work during the third impulse. Conversely, at high speeds the CoM path is so shallow that the forward‐inclined impulse contributes a high positive work that is dissipated with the third impulse. CoM: center of mass [Color figure can be viewed at wileyonlinelibrary.com]

The principles of collision geometry introduced for quadrupedal gaits by Ruina et al., (2005) demonstrate that more, smaller deflections in center of mass velocity results in lower positive work demands than fewer, larger deflections resulting in the same overall change. A gait with more impulses each stride may have the capacity to reduce the CoM work demand, but only if the impulses and CoM velocity vectors can be appropriately orientated, and this detail accounts for the changes in optimal gait found as a function of speed (and gravity and stride period). At low speeds and impulse inclinations, trot is found to require less work than canter (Figure 3b), despite having only two impulses per stride as opposed to the canter's three. The same is predicted at very high speeds; canter is only favored over a bounded range of speeds for a given impulse orientation (Figure 3). Under only these conditions are the impulses orientated close to perpendicular to the CoM trajectory, such that each change in velocity involves both a decrease and increase in magnitude; such that *V*_min_ falls somewhere between *U* and *V* for each impulse (Figure 4) and each collision is “pseudoelastic.” For canter at too‐declined a CoM trajectory (as at low speeds), the impulse from the single hindlimb only dissipates energy, and the impulse from the single forelimb provides only positive work associated with propelling the CoM upwards (Figure 5a). At too‐shallow a CoM incidence (as at high speeds), the opposite is the case, with the impulse from the single hindlimb producing only positive work, largely to propel the CoM forwards (Figure 5b). In these two cases, the conditions for a “pseudoelastic” collision (see Ruina et al., 2005) are only met for the middle, paired, vertical impulse. A collisional principle introduced by Ruina et al. (2005) is pertinent here: “even with no elastic recovery, it is energetically beneficial to make every collision a pseudoelastic collision” (p. 178).

**Figure 6:**
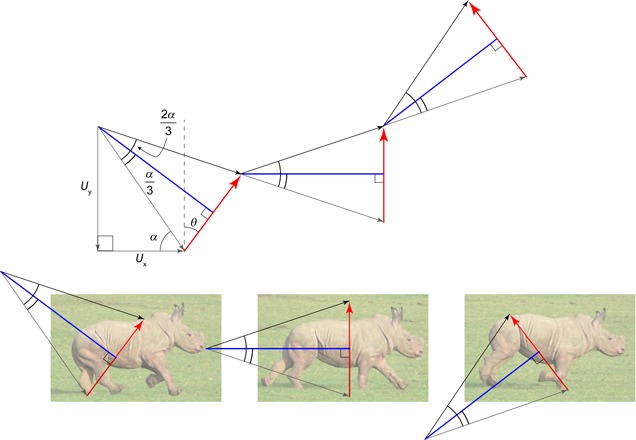
The velocity/impulse geometry of an idealized canter with each of three pseudoelastic impulses (hind, hind–fore pair, fore) acting perpendicular to *V*_min_ in each case allows an analytical approximation for conditions good for cantering. The angle *α* depends on the initial horizontal velocity (*U_x_* = ${\bar{V}}_{x}$) and the vertical velocity resulting from the ballistic flight phase (greater with higher *T*_stride_). The center of mass (CoM) path must be altered by 2*α* (downwards to up) over the course of the three impulses acting at +*θ*, vertically and –*θ*. Very high values of *α* and *θ* are shown for clarity. Cantering should be effective if $\hat{V_{\text{x}}}\approx \frac{\hat{T_{\text{stride}}}}{2\tan\left(\frac{3\theta }{2}\right)}$ [Color figure can be viewed at wileyonlinelibrary.com]

Approximate analytical conditions for effective cantering can be derived assuming work is low if *V*_min_ falls half way between *U* and *V* for each of the three impulses. In this approximation, it is assumed that the three impulses split weight support evenly. This simplifies the necessary geometry (Figure 6), and finds a prediction that falls within the region determined by the numerical model, but deviates considerably from observed loading distributions between limbs (Merkens et al., 1993). The center of mass begins the series of three canter collisions after a period of ballistic flight, so with a downward vertical velocity of

(15) $$U_{\text{y}}=-\frac{gT_{\text{stride}}}{2}.$$

The initial CoM velocity angle to the horizontal *α*, where the horizontal velocity *U*_*x*_ equals the mean horizontal velocity, is:

(16) $$\alpha \;=\;\tan^{-1}\left(\frac{U_{y}}{U_{x}}\right).$$

To return the CoM to an upward trajectory before the next ballistic phase, it has to be redirected through an angle of 2*α* over the course of three impulses. If each impulse is perfectly pseudoelastic *V*_min_ bisects between the initial and final velocity of each stage, and the geometry of Figure 6 can be rearranged to give:

(17) $$\theta =\frac{2}{3}\tan^{-1}\left(\frac{\hat{T_{\text{stride}}}}{2\hat{V_{\text{x}}}}\right)$$

or

(18) $$\hat{V_{x}}\;=\;\frac{\hat{T_{\text{stride}}}}{2\;\tan\;\left(\frac{3\theta }{2}\right)}.$$

This relationship is shown with the numerical results (Figure 3a).

## 11 THE GATHERED 4‐BEAT GALLOP

The results for the gathered gallop can be understood from the principles discussed above. With the two impulses from the hindlimbs being identical, and acting at functionally the same instant, they can be treated as a single impulse of double magnitude. The same applies to the two impulses from the forelimbs. The best that can be imagined, then, is that the two (combined) impulses of the gathered gallop achieve the same work demand as the two impulses per stride of the trot. This is approached at the trough of Figure 3b—when the ballistics of the aerial phase align the CoM trajectory appropriately (near‐perpendicularly) with the impulse vectors.

## 12 THE EVEN 4‐BEAT GALLOP

From observation of terrestrial mammals locomoting at progressively higher speeds, it would be expected that, beyond the canter, some form of gallop might be predicted to be work‐minimizing. This is found to be untrue for the idealized gaits surveyed here. Does this merely reflect the limited range of impulsive gaits being considered here? No, or at least not for the limiting case of very high speeds. For any gait with inclined impulses and sufficiently high velocity, the shallow center of mass trajectory results in each inclined impulse becoming entirely work‐generating (if the impulse is forward) or work‐absorbing (if the impulse is backward) and not pseudoelastic, simplifying the calculations for mechanical work (see Ruina et al., 2005; Appendix A1) and allowing their horizontal and vertical components to be treated separately. The work required for an impulse scales with the velocity in the direction of the impulse. Inclined impulses at higher velocities therefore demand progressively higher mechanical work associated with fluctuations in horizontal velocity, whereas the purely vertical impulses of idealized trot are not influenced by horizontal velocity and the work requirements are only those required to provide the vertical ballistic motions (Equation (1)). This indicates that the result found for the selected idealized gaits will stand for any comparison between trot and a gait with inclined impulses: at some high velocity and shallow CoM trajectory, impulsive trotting would again become the work‐minimizing gait.

## 13 GENERAL DISCUSSION

The approach taken here, of considering work‐minimizing gaits for animals experiencing momentary impulses at each end of a stiff back, about a point center of mass, clearly departs considerably from reality. However, it does provide a first account based on the fundamentals of work minimization for (a) the prevalence of trotting (if $\hat{I}$ < 1), (b) the scarcity of tolt (if $\hat{I}$ < 1), and (c) the transition towards a canter with *increasing* speed due to the benefit of three impulses per stride achievable with only a limited range of CoM trajectories at the end of the ballistic aerial phase.

Which aspects of the model reductions and assumptions should be questioned in attempts to account for what this approach fails to explain? For instance, what accounts for the transition to gaits other than trot at the highest speeds?

### 13.1 Places to start might include

● Intermediate $\hat{I}$. This enables net impulse vectors to be orientated in directions other than through the center of mass. It may allow exploration of the trade‐offs between minimizing the energetic costs of pitching versus CoM work minimization, and how these might scale with speed. Reported empirical impulse inclinations are far lower than those that would result in zero‐pitch moments (they would not pass through the CoM)—for instance, Merkens et al. (1993) report approximately 6° for cantering horses rather than the required 27–30° required (Williams, Tan, Usherwood, & Wilson, 2009)—potentially reflecting this compromise.
● Variation of $\hat{I}$. There is no reason for $\hat{I}$ to remain constant across gait and speed, especially for animals with flexible backs (greyhound, cheetah, etc.).
● Finite stance periods and fluctuating force vectors. Alexander's models pioneered such models applied to bipedal and quadrupedal gaits (e.g., Alexander, 1980, 1984); the challenge is often the matter of establishing appropriate constants for fair gait comparisons.
● Costs other than pure mechanical work. Peak bone stress has been associated with the trot‐gallop transition (Biewener & Taylor, 1986; Rubin & Lanyon, 1982), but whether this should be viewed as a cue, a fundamental cause, or merely a correlate of gait transition is unclear. In terms of energetics, one notable alternative cost that may be particularly relevant to small and very fast animals is peak mechanical power: if there is a cost to activating a volume of muscle, and muscle must be activated to provide mechanical power, gait strategies that limit peak power may be favored even if they require a little more mechanical work (Usherwood, 2016; Usherwood, Hubel, Smith, Self Davies, & Sobota, 2018).

To conclude, considerations of highly reductionist work‐minimizing models continue to provide insight into the fundamental principles underlying gait mechanics and gait selection. However, an elegant mechanistic account for trot, canter, and gallop gaits and their transitions with speed remains elusive.
